# GATA3 expression in clear cell adenocarcinoma of the lower urinary tract: a potential diagnostic pitfall

**DOI:** 10.1186/s13000-022-01269-6

**Published:** 2022-11-01

**Authors:** Mahmut Akgul, Robert Humble, Abdullah Osme, Servet Yuce, Elif N. Kocak, Parisa Najafzadeh, Ankur Sangoi, Niharika Pattnaik, Sourav Mishra, Shivani Sharma, Nada Shaker, Seema Kaushal, Manas Baisakh, Andrea R. Lightle, Bonnie L. Balzer, Guang-Qian Xiao, Gregory T. MacLennan, Adeboye O. Osunkoya, Anil Parwani, Liang Cheng, Andrew Bellizzi, Sambit K. Mohanty

**Affiliations:** 1 Department of Pathology and Laboratory Medicine, Albany Medical Center, Albany, NY, USA; 2 Department of Pathology and Laboratory Medicine, University of Iowa, Iowa City, IA, USA; 3 Department of Pathology and Laboratory Medicine, University Hospitals Cleveland Medical Center, Cleveland, OH, USA; 4 Department of Public Health, Istanbul University School of Medicine, Istanbul, Turkey; 5 Department of Pathology, Keck School of Medicine, University of Southern California, Los Angeles, CA, USA; 6 Department of Pathology, El Camino Hospital, Mountain View, CA, USA; 7 SRL Diagnostics, Bhubaneswar, Orissa, India; 8 Apollo Hospitals, Bhubaneswar, Orissa, India; 9 DCP, Core Diagnostics, Gurgaon, Haryana, India; 10 Department of Pathology and Laboratory Medicine, The Ohio State University Wexner Medical Center, Columbus, OH, USA; 11 AIIMS, Delhi, India; 12 Department of Pathology, Cedars-Sinai Hospital, Los Angeles, CA, USA; 13 Department of Pathology and Laboratory Medicine, Emory University, Atlanta, GA, USA; 14 Department of Pathology and Urology, Indiana University, Indianapolis, IN, USA; 15 Oncologic Surgical and Molecular Pathology, Advanced Medical Research Institute, Senior Oncologic Surgical and Molecular Pathologist, CORE Diagnostics, 406, Udyog Vihar III, 122001 Gurgaon, Haryana, India

**Keywords:** GATA3, Immunohistochemistry, Clear cell adenocarcinoma, Lower urinary tract

## Abstract

**Background:**

Clear cell adenocarcinoma of the lower urinary tract (CCACLUT) is a rare primary malignant neoplasm with heterogenous morphology. There is a paucity of data in the literature regarding its immunohistochemical profile.

**Methods:**

The immunohistochemical features (extent and intensity) of a multinational cohort of CCACLUT were evaluated with comparison between clear cell adenocarcinoma of the female genital tract (CCACFGT, tissue microarray) and nephrogenic adenoma (NA).

**Results:**

33 CCACLUT (24 female, 9 male; mean age 59 years) were collected. CCACLUT most commonly arose from the urinary bladder (26/33, 78%), particularly from the trigone (10/33, 30.3%) followed by the urethra (8/33, 22%). All 12 NA cases were located at the urinary bladder, whereas the most common CCACFGT location was the ovary (29/56, 52%). None of the CCACLUT patients had, intestinal metaplasia, NA, or urothelial carcinoma. One patient had concurrent endometriosis of the sigmoid colon. Most frequently observed morphology in CCACLUT was papillary/tubulocystic (9/3; 27.3%), followed by papillary/tubular (6/33; 18.2%) and papillary/solid (5/33; 15.2%). GATA3 expression was significantly higher in CCACLUT (18/33, 54.5%) and NA (6/12, 50%), when compared to CCACFGT cases 6/56, 11.7%)(p = 0.001 and p = 0.022, respectively). The extent of GATA3 was significantly higher in CCACLUT group (19.2 ± 16.6%) than the other groups (9.6 ± 22.5% in NA and 2.6 ± 9% in CCACFGT group) (p = 0.001). 4/33 patients (12.1) had weak, 10/33 patients (30.3%) had moderate, and 4/33 patients (12.1%) had strong GATA3 intensity in CCACLUT group. In NA group, one patient (8.3%, 1/12) had weak, one patient (8.3%, 1/12) had moderate and 4 patients (33.3%, 4/12) had strong GATA3 intensity. Most cases (CCACLUT 29/33, 88%; NA 11/12, 92%; CCACFGT 46/56, 82.1%) had positive Napsin A expression, by which CCACLUT had significantly more cases with Napsin A expression (p = 0.034). p63 was consistently negative in all cases (30/33 (91.9%) CCACLUT; 12/12 (100%) NA; 42/56 (75%) CCACFGT. Ki67 (MIB) proliferation index was significantly higher in CCACLUT group (54.6 ± 21%) when compared to NA group (4.5 ± 2.7%) and CCACFGT group (35.5 ± 25.8%) (p = 0.001).

**Conclusion:**

CCACLUT has consistent GATA3 expression, which may cause challenge in the diagnosis of urothelial carcinoma but can be used to distinguish CCACLUT from CCACFGT.

## Highlights

Clear cell adenocarcinoma of the lower urinary tract is rare, with heterogenous morphology.

GATA3 expression can be seen in these tumors, causing diagnostic challenge in the urinary tract or in the metastatic setting.

Along with morphologic clues, immunohistochemical panel of PAX8, GATA3, and p63 might be useful to distinguish these tumors from urothelial carcinoma.

## Introduction

**Fig. 1:**
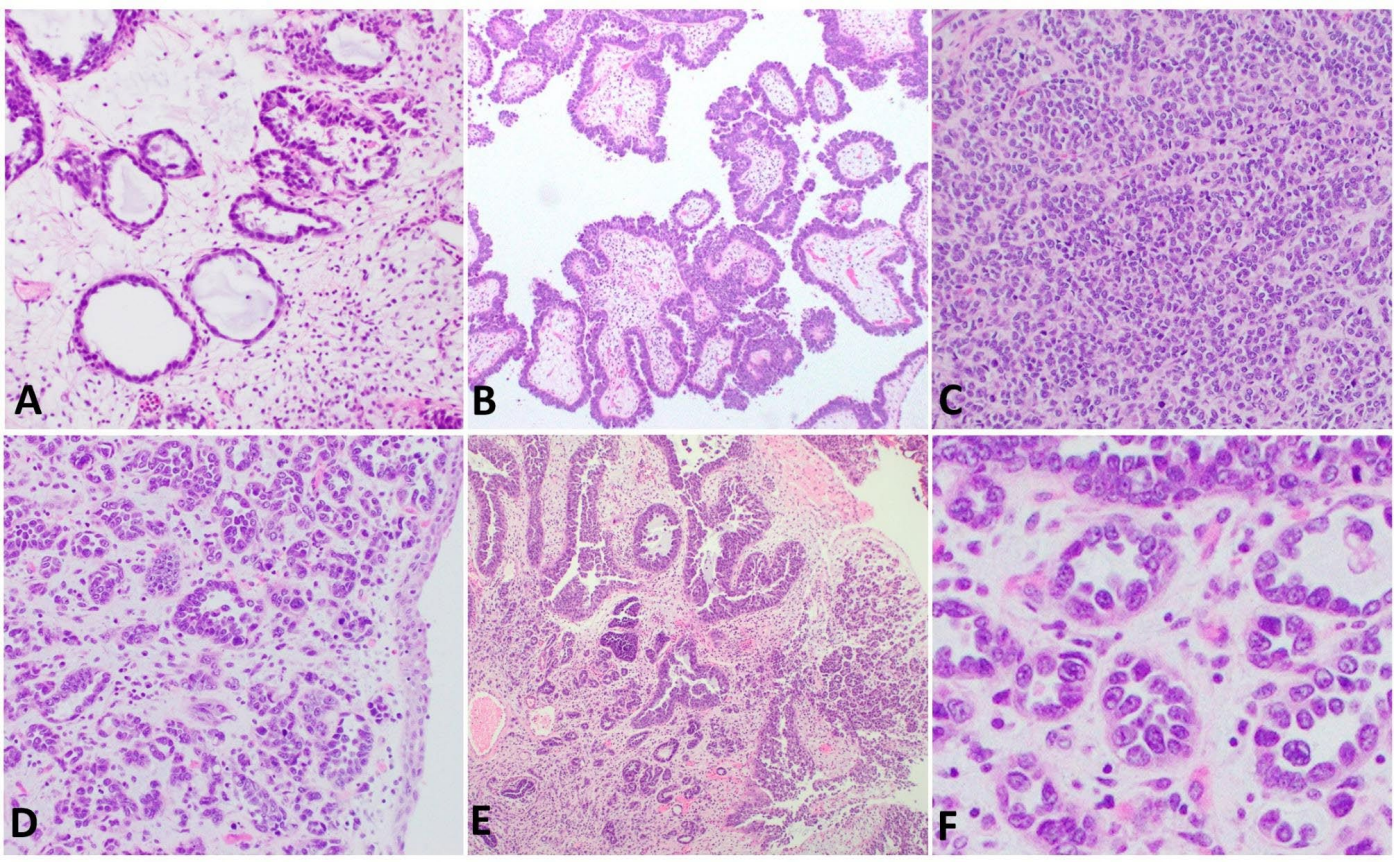
Clear cell adenocarcinoma of the lower urinary tract demonstrates various morphologic patterns including tubulocystic (A; 100X), papillary (B; 100X), diffuse (C; 100X), or nephrogenic adenoma-like morphology (D; 100X); multiple patterns are often present (E; 40X). High-grade cytologic atypia is almost always observed, with characteristic “hobnailing” of the tumor cells (F; 200X)

Clear cell adenocarcinoma (CCAC) is a malignant neoplasm arising predominantly in the female genital tract (FGT), particularly from the ovary [1] as well as a variant of endometrial carcinoma [2]. CCAC can rarely be seen primarily in the lower urinary tract (LUT)[3], and most commonly occurs in the urethra (particularly in diverticula) and trigone or posterior wall of the urinary bladder [4], with significant female predominance (female-to-male ratio = 3:1) [3, 4], although occurrence in male LUT is well documented [5]. CCAC may demonstrate tubulocystic (Fig. 1 A), papillary (Fig. 1B), or diffuse growth patterns (Fig. 1 C) [3]. Occasionally nephrogenic adenoma-like (NA-like) morphology (Fig. 1D) may be present[3]. These growth patterns are often observed in the same tumor with varying proportions (Fig. 1E), and high-grade cytologic atypia is almost always present (with descriptive cellular “hobnailing”; Fig. 1 F). Concurrent endometriosis and/or urothelial carcinoma (UCa) in a subset of cases are reported [6]. This morphologic heterogeneity along with additional neoplastic and non-neoplastic findings cause challenge in the differential diagnosis, mimicking UCa, particularly of glycogen rich (“clear cell”) subtype [7] and/or NA. The current view of the World Health Organization (WHO) positions these tumors under “tumors of the Müllerian type” [3], due to its morphologic features resembling its FGT counterpart as well as the persistent expression of PAX8; accompanied by keratin 7, napsin A, and HNF1B, further supporting the Müllerian differentiation. However, there is conflicting evidence whether these tumors originate from non-urothelial cell type such as some embryologic Mullerian remnants or derivation from urothelial carcinoma [8].

GATA3 is one of the six members of the *GATA* gene family of transcription factors and is first identified in the hematopoietic system, particularly in T-helper type 2 cells [9]. GATA3 takes part in the development of various tissue types such as T-cells, skin, and breast parenchyma [10]. GATA3 expression is vastly utilized in the breast and the urothelial carcinomas due to its high specificity; and many other benign and malignant lesions arising from skin, kidney, uterus, testis, ovary, and pancreas [11]. UCa shares its GATA3 expression with NA [12] and paraganglioma arising from the urinary tract, although GATA3 expression in CCAC of the urinary tract has not been evaluated.

## Methods

### Case acquisition

After approval of the local institutional review boards, multi-insitutional cohort of CCAC of the LUT cases (CCACLUT) were collected. Patients’ gender, age at the diagnosis, procedure (transurethral resection (TUR) and/or cystectomy/cystoprostatectomy), tumor site, and gross tumor size (cm) were recorded. Hematoxylin and eosin (HE) stained slides of the tumor sections were reviewed by pathologists with genitourinary pathology expertise (M.A., S.M., A.O., L.C., G.T.M, A.S., G.Q.X., A.B.) and the diagnoses were confirmed. Predominant morphologic features were noted. Similarly, NA and CCAC of the FGT (CCCFGT) cases were collected from the archives of department of pathology and laboratory medicine at University of Iowa were gathered. For CCACFGT cases, tissue microarrays (TMAs) were constructed with Manual Tissue Arrayer MTA-1 (Beecher Instruments Inc.) utilizing 1 mm punches (Estigen OÜ), with each tumor sampled in triplicate. Immunohistochemical (IHC) assays with appropriate controls including GATA3, p63, keratin 7, napsin A, and MIB1 (Ki-67) were selected for evaluation. Intensity of the IHC was quantified in the spectrum of 0–3 (0 = no expression; 1 = weak; 2 = moderate; 3 = strong), whereas tumor extent was quantified as percentage of tumor cells expressing the biomarker. The H-score was determined by adding the results of multiplication of the percentage of cells with staining intensity ordinal value (scored from 0 for “no signal” to 3 for “strong signal”) with 300 possible values.

### Statistical analysis

Data analysis was performed using SPSS (Statistical Package for Social Sciences; SPSS Inc., Chicago, IL) version 25.0. Descriptive data were shown as numbers (n) and percentage (%) in categorical data and mean ± standard deviation (mean ± SD) in continuous data. Pearson Chi-square test was used to compare categorical variables between groups. Conformity of continuous variables to normal distribution was evaluated with Kolmogorov-Smirnov test. Mann Whitney U-test was used to compare normally distributed variables in two groups. One Way ANOVA test was used for parametric variables and Kruskal Wallis test was used for nonparametric variables when comparing more than two groups. Bonferroni correction was used for pair-group comparison in post-hoc analyses. Spearman correlation analysis was used to compare two continuous variables. p < 0.05 was accepted as statistically significant.

## Results

### Cohort characteristics

A total of 101 patients were included in this study, including 33 patients with CCAC, 12 with NA, and 56 with CCACFGT. In the CCACLUT group, 24 (73%) patients were female and 9 (27%) were male. 4 of 12 patients (%33) in NA group were female. The mean age of the patients in CCACLUT group was 59 years, while it was 64 years in NA group, and 64 years in CCACFGT. In 4/33 (12.1%) patients with CCACLUT, tumor exclusively arose from the urethra, whereas 26/33 (78.7%) patients tumor arose from the urinary bladder. In 3/33 (9%) patients, both urethra and urinary bladder were involved. Tumor size was available from 27 patients (range 1.7–9 cm) in the CCACLUT group and 50 patients (1.2–27.4 cm) in the CCACFGT group. CCACLUT most commonly arose from trigone (10/33, 30.3%). CCACFGT mainly arose from ovary (29/56; 52%), followed by endometrium (23/56; 41) and cervix (4/56; 7%). Most frequently observed morphology in CCACLUT was papillary/tubulocystic (9/33; 27.3%), followed by papillary/tubular (6/33; 18.2%) and papillary/solid (5/33; 15.2%). No cases had history of the endometriosis in the CCACLUT cases, one case had concurrent endometriosis identified in the sigmoid colon. None of the CCACLUT cases had intestinal metaplasia, NA, or UCa in the urinary tract. Table 1 includes cohort characteristics.

**Table 1:** Clinicopathologic characteristics of 101 participants

		Count	%
Group	CCACLUT	33	32.7
	NA	12	11.9
	CCACFGT	56	55.4
	Total	101	100
Gender	Female	84	83.2
	Male	17	16.8
	Total	101	100
Age, year		101	Mean 63 ± 14
Size, cm		77	Mean 8 ± 6
Location	Bladder	39	38.6
	N/A	6	5.9
	Endometrium	23	22.8
	Ovary	29	28.7
	Cervix	4	4.0
	Total	101	100
Procedure type	Hysterectomy with BSO	25	24.8
	Salpingo-oophorectomy	22	21.8
	Cystectomy	18	17.8
	TURBT	8	7.9
	Cystoprostatectomy	7	6.9
	Biopsy	6	5.9
	Transurethral resection	3	3.0
	Hysterectomy	3	3.0
	Oophorectomy	2	2.0
	Anterior pelvic exenteration	1	1.0
	Cystectomy with hysterectomy	1	1
	Hysterectomy with bilateral salpingectomy and right oophorectomy	1	1
	Hysterectomy with left salpingo-oophorectomy	1	1
	Radical cystoprostatectomy	1	1
	Radical hysterectomy	1	1
	Total pelvic exenteration	1	1
	Total	101	100
Predominant morphology	Tubulocystic/Papillary	15	14.9
	Papillary	13	12.9
	Solid	12	11.9
	Papillary/Tubulocystic	9	8.9
	Tubulocystic	8	7.9
	Papillary/Tubular	6	5.9
	Solid/Tubulocystic	6	5.9
	Tubulocystic/Solid	6	5.9
	Papillary/Solid	5	5
	Tubular/glandular	5	5
	Diffuse/tubulocystic	4	4
	Papillary/Tubulocystic/Solid	3	3
	Solid/Papillary	3	3
	Tubular/Tubulocystic	2	2
	Cystic	1	1
	Solid/Papillary/Tubulocystic	1	1
	Solid/Tubulocystic/Papillary	1	1
	Tubular/Papillary	1	1
	Total	101	100

*Abbreviations; CCACLUT: Clear Cell Adenocarcinoma of Lower Urinary Tract, NA: Nephrogenic adenoma, CCACFGT: Clear Cell Adenocarcinoma of Female Genital Tract*

### GATA3 expression

**Fig. 2:**
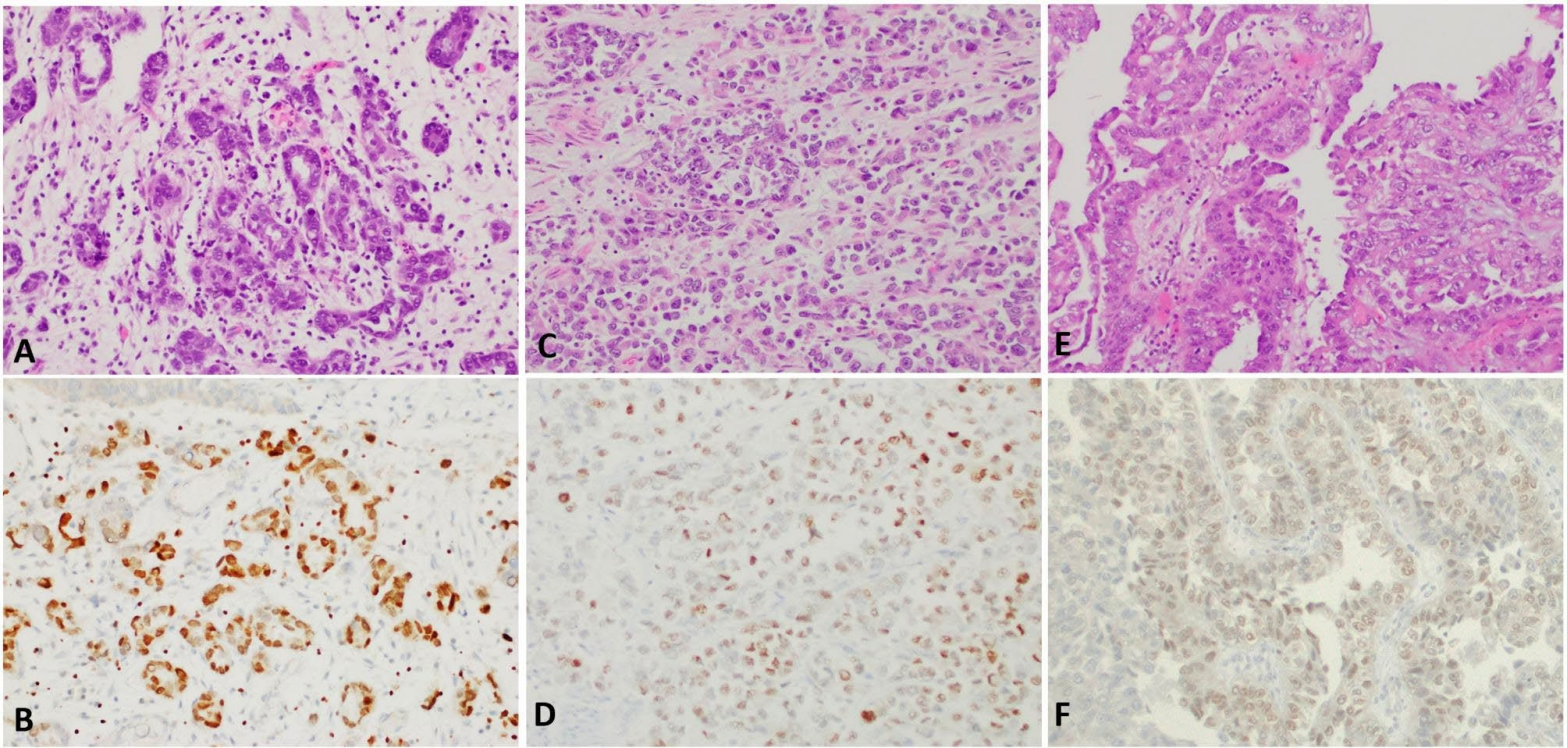
GATA3 shows various expression intensity in the clear cell adenocarcinoma of the lower urinary tract including strong (2 A-2B; 200X), moderate (2 C-2D; 200X); and weak (2E-2 F; 200X).

**Fig. 3:**
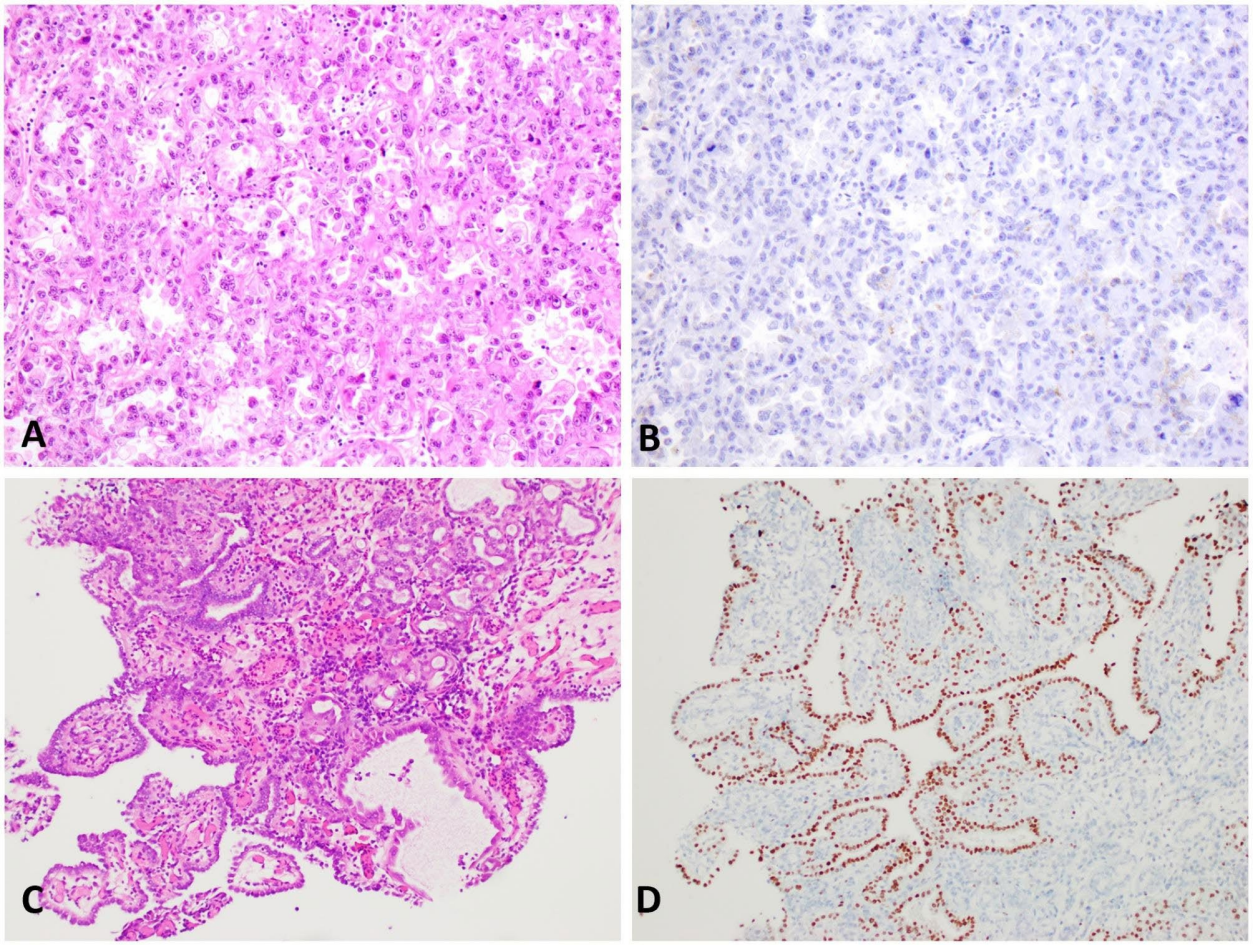
Majority of clear cell adenocarcinoma of the female genital tract is negative for GATA3 (ovarian primary; 3 A-3B; 200X); in contrast, frequent GATA3 expression is present in nephrogenic adenoma (3 C-3D; 200X).

More than half of the CCACLUT (18/33, 54.5%) and half of the NA cases (6/12, 50%) had GATA3 expression. Four of 33 (12.1%) CCACLUT patients had strong GATA3 expression (Fig. 2A-2B). About one-third (10/33, 30.3%,) of the patients in CCACLUT group had moderate GATA3 expression (Fig. 2C-D), while four patients had weak (4/33, 12.1%) expression (Fig. 2E-F). In contrast, most of the CCACFGT cases were negative (50/56, 89.3%) for GATA3 (Fig. 3A-3B). There was significant difference between CCACFGT and NA groups (p = 0.022), and between CCACLUT and CCACFGT groups (p = 0.001) in terms of number of cases with GATA3 expression, although there was no statistically significant difference between NA and CCACLUT groups (p = 0.254) (Table 2). The extent of GATA3 was significantly higher in CCACLUT group (19.2 ± 16.6%) than the other groups (9.6 ± 22.5% in NA and 2.6 ± 9% in CCACFGT group) (p = 0.001). Finally, in CCAFGT group, 1 patient (1.7%, 1/56) had weak, 2/56 patients (3.4%) had moderate, and 3/56 patients (5.1%) had strong GATA3 intensity. In terms of intensity, the difference was statistically significant between CCACFGT and NA groups (p = 0.022), and between CCACLUT and CCACFGT groups (p = 0.001) while the difference was not significant between NA and CCACLUT groups (p = 0.254).

**Table 2:** Staining results of various antibodies on immunohistochemistry in CCACLUT, NA and CCACFGT groups.

		CCACLUT		NA		CCACFGT		Total		
		Count	%	Count	%	Count	%	Count	%	P value
PAX8	Negative	0		0		1	1.8	1	1	0,231^1^
	Weak	0		0		0		0		
	Moderate	2	7.1	0		0		2	2.1	
	Strong	26	92.9	12	100	55	98.2	93	96.9	
	Total	28	100	12	100	56	100	96	100	
P63	Negative	30	90.9	12	100	42	75	84	83.2	0,101^1^
	Weak	0		0		8	14.3	8	7.9	
	Moderate	3	9.1	0		4	7.1	7	6.9	
	Strong	0		0		2	3.6	2	2	
	Total	33	100	12	100	56	100	101	100	
Napsin A	Negative	4	12.1	1	8.3	10	17.9	15	14.9	**0,006** ^**1**^
	Weak	7	21.2	0		7	12.5	14	13.9	
	Moderate	7	21.2	0		1	1.8	8	7.9	
	Strong	15	45.5	11	91.7	38	67.9	64	63.4	
	Total	33	100	12	100	56	100	101	100	
GATA3	Negative	15	45.5	6	50	50	89.3	71	70.3	**0,001** ^**1**^
	Weak	4	12.1	1	8.3	1	1.8	6	5.9	
	Moderate	10	30.3	1	8.3	2	3.6	13	12.9	
	Strong	4	12.1	4	33.3	3	5.4	11	10.9	
	Total	33	100	12	100	56	100	101	100	

^1^Pearson Chi-Square test

*Abbreviations; CCACLUT: Clear Cell Adenocarcinoma of Lower Urinary Tract, NA: Nephrogenic adenoma, CCACFGT: Clear Cell Adenocarcinoma of Female Genital Tract*

**Fig. 4:**
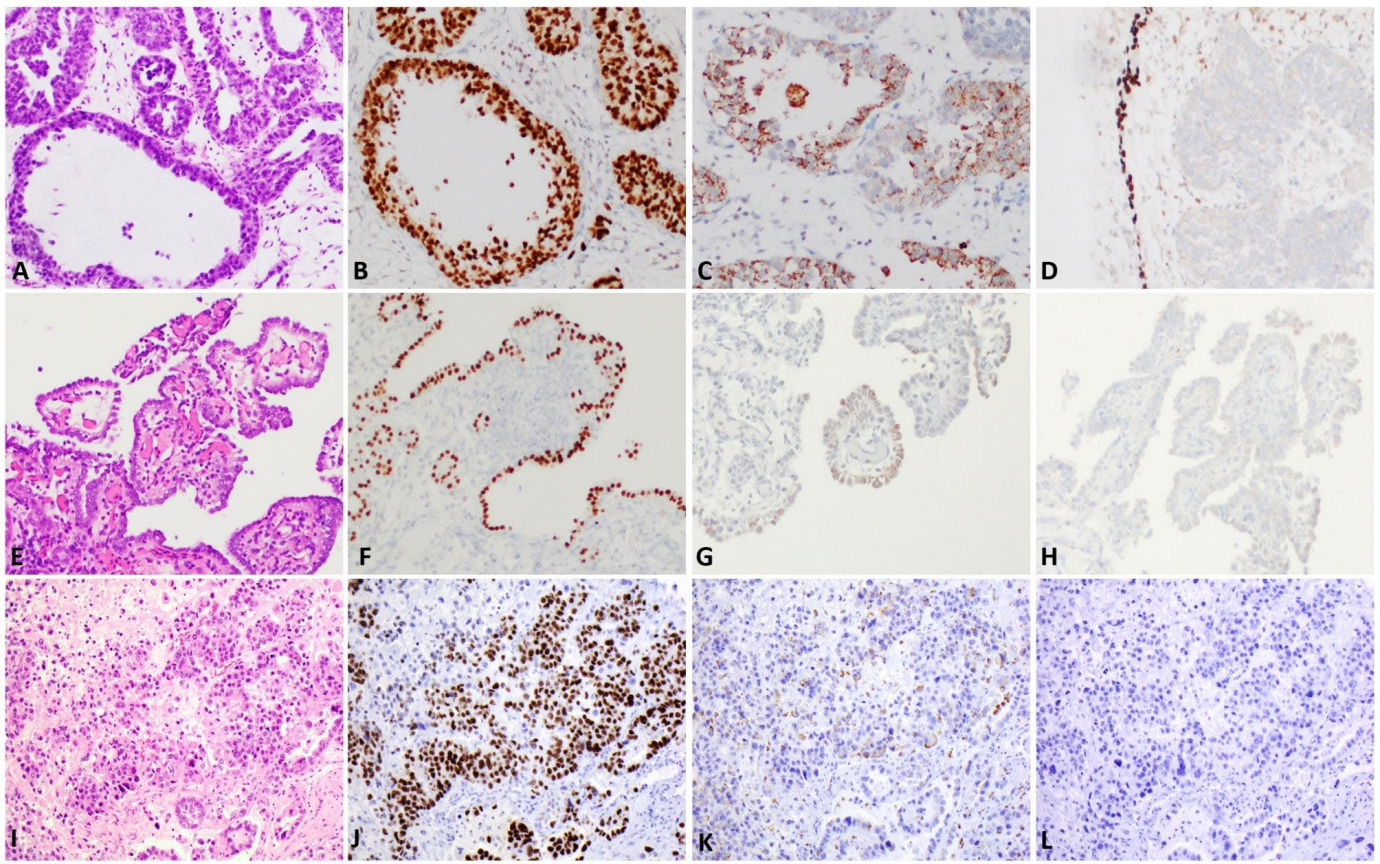
PAX8 expression is strong and diffuse in clear cell adenocarcinoma of lower urinary tract (CCACLUT; 4 A-4B; 200X), nephrogenic adenoma (4E-4 F; 200X), and clear cell adenocarcinoma of the female genital tract (CCACFGT; ovarian primary; 4I-4 J; 200X). Somewhat patchy but persistent Napsin A was present in CCACLUT (4 C; 200X), NA (4G; 200X), and CCACFGT (4 K; 200X). p63 was all negative in CCACLUT (4D; base of the urothelium as control; 200X) and NA (4 H; 200X); as well as all but one CCACFGT cases (4 L; 200X).

The mean H score of GATA3 was statistically significantly higher in the CCACLUT group (28.9 + 36.1) than in the CCACFGT group (6.9 + 26.5) but was similar with NA group (27.1 + 67.8) (p: 0.001).

### PAX8 expression

All CCACLUT and NA cases diffusely express PAX8 with predominantly strong (26/28; 92.9%; Fig. 4A-4B) and exclusively strong intensity (12/12; 100%; ), respectively. Except for one case (55/56; 98%), all CCACFGT cases had diffuse and strong PAX8 expression.

### Napsin A expression

Most cases in all groups (CCACLUT 29/33, 87.9%; NA 11/12, 91.7%; CCACFGT 46/56, 82.1%) had positive Napsin A expression. Napsin A extent was 42.2 ± 30.7% in CCACLUT group; 36.6 ± 30% in NA group; and 25.8 ± 30.2 in CCACFGT group. Napsin A extent was significantly higher at CCACLUT group than the other groups (p = 0.034). Strong Napsin A expression was seen in 15/33 (45.5%), 11/12 (91.7%), and 38/56 (67.9%) in CCACLUT, NA, and CCACFGT, respectively, which reached statistical significance between CCACLUT and NA (p:0,041) and between CCACLUT and CCACFGT groups (p = 0.007) (Table 2).

### P63 expression

Only 3/33 (9.1%) CCACLUT cases were positive for p63 with moderate intensity, and none of the NA cases expressed p63 (0/12). 14/56 (25%) of CCACFGT cases were positive for p63 with mostly weak intensity (8/56; 14.2%). There was no significant difference between CCACLUT, NA and CCACFGT groups in terms of p63 intensity (p: 0.101).

### MIB1 (Ki67) expression

MIB extent, also known as proliferation index, was highest at CCACLUT group (54.6 ± 21%) which significantly higher than NA group (4.5 ± 2.7%) and CCACFGT group (35.5 ± 25.8%) (p = 0.001).

### Other biomarkers

Keratin 7, AMACR, and WT1 biomarkers were only examined in CCACLUT group. Keratin 7 expression was present in almost all available (28/29, 97%) CCACLUT cases, with 22/25 cases having more than 50% positive cells (88%) and strong expression in 20/29 cases (69%). Similarly, AMACR was positive in most of the available CCACLUT cases (25/27, 93%). WT1 was available in 22/33 CCACLUT cases and all were negative.

## Discussion

Few CCACLUT cases have been documented in the literature with scarce data on the immunoprofile. Young and Scully reported 3 CCAC of the urinary bladder with review of 16 CCACLUT in the pre-IHC era, followed by Oliva and Young’s [13] report on exclusively urethral CCAC of 19 mostly female (n = 18) patients, establishing morphologic features and drawing close association to the urethral diverticulum and lack of endometriosis. Early reports on CCACLUT IHC highlighted the striking overlap between top differential entities including UCa, NA, and CCACFGT; all expressing non-specific pancytokeratin, CAM5.2, keratin 7, and EMA markers [4]. Although there has been lack of dedicated large CCACLUT studies on practical markers; case reports and small series showed that CCACLUT express AMACR [8], PAX8 [14], and napsin A [12]. Our study not only contains the largest multi-institutional CCACLUT cohort but also claims to be the first report evaluating GATA3 IHC expression in these tumors. GATA3 expression was present in more than half of the CCACLUT cases with significantly higher extent and intensity when compared to CCACFGT and NA, which proves the presence of the concerning overlap between CCACLUT and UCa. Morphologic heterogeneity coupled with persistent GATA3, and keratin 7 expression may cause the misdiagnosis of CCACLUT as UCa. Our study also found persistent lack of p63 expression in CCACLUT, which may be helpful in differentiating from UCa, as p63 is strongly positive in most of the UCa [15].

The controversy on the cellular origin of CCACLUT remains despite several reports dedicated solely to address the issue [8, 16]. Oliva et al [6] reported four CCACLUT with concurrent UCa, with additional five cases demonstrating “pseudostratified epithelium reminiscent of transitional epithelium”, presenting as supporting evidence of the urothelial origin of CCACLUT. However, there were also four CCACLUT in the same cohort with concurrent Müllerian-type tissue. Sung et al [8] found chromosome number alterations (gains on chromosome 3, 7, and 17 in all 12 CCACLUT cases; 9p21 loss in 3/12 cases) using UroVysion fluorescence in situ hybridization (FISH) assay, similar to UCa. In contrast, Ortiz-Bruchle found *ARID1A* gene mutations in 5/11 CCACLUT cases with no *TERT* gene promoter alterations [17]. Similarly, our recent report on comprehensive next generation sequencing (NGS) in one of the cases in the current cohort [18] identified *ARID1A, PBRM1, ERBB4, and SMARCA4* mutations. Another hypothesis is the malignant transformation of NA to CCACLUT, suggested in two separate case reports [19, 20] [1]. The lack of concurrent NA, endometriosis in the urinary bladder, or UCa, and consistent expression of biomarkers associated with the Mullerian origin in our cohort further support that CCAC of the urinary tract does not arise from the urothelium, which is in line with the current WHO designation of the CCAC of the urinary tract as non-urothelial (under tumors of the Mullerian origin).

There are several limitations in our study. Multiple labs and different clones were involved in the IHC evaluation of this multinational cohort, causing comparison of potentially different assay performances. We did not include a group of UCa in this study, although morphologic and biomarker characteristics of UCa and its subtypes are well established [15]. CCACFGT cohort was included in the form of TMA that prevented evaluation of more tissue. Finally, we did not include the outcome data of these patients in our study, therefore, we have no data with regards to GATA3 expression and its impact on the survival in CCACLUT or CCACFGT.

In conclusion, CCACLUT has consistent GATA3 expression is a subset of cases, which may cause misinterpretation as UCa, but can be used to distinguish CCACFGT or NA. A limited panel of IHC including PAX8, GATA3, and p63 in suspected cases may prevent potential misdiagnosis. Studies with comprehensive molecular interrogation is needed to further characterize these tumors.

## Data Availability

Study data is available upon request. Please contact the corresponding author.
